# Methods and rationale of the DISCOVER CKD global observational study

**DOI:** 10.1093/ckj/sfab046

**Published:** 2021-04-11

**Authors:** Roberto Pecoits-Filho, Glen James, Juan Jesus Carrero, Eric Wittbrodt, Steven Fishbane, Alyshah Abdul Sultan, Hiddo J L Heerspink, Katarina Hedman, Eiichiro Kanda, Hungta (Tony) Chen, Naoki Kashihara, James Sloand, Mikhail Kosiborod, Supriya Kumar, Mitja Lainscak, Matthew Arnold, Carolyn S P Lam, Björn Holmqvist, Carol Pollock, Peter Fenici, Peter Stenvinkel, Jennie Medin, David C Wheeler

**Affiliations:** 1 School of Medicine, Pontifícia Universidade Católica do Paraná, Curitiba, Brazil; 2 Arbor Research Collaborative for Health, Ann Arbor, MI, USA; 3 AstraZeneca, Cambridge, UK; 4 Department of Medical Epidemiology and Biostatistics, Karolinska Institutet, Stockholm, Sweden; 5 AstraZeneca, Gaithersburg, MD, USA; 6 Division of Nephrology, Zucker School of Medicine at Hofstra/Northwell, Hempstead, NY, USA; 7 Department of Clinical Pharmacy and Pharmacology, University of Groningen, Groningen, the Netherlands; 8 AstraZeneca, Gothenburg, Sweden; 9 Medical Science, Kawasaki Medical School, Kurashiki, Japan; 10 Department of Nephrology and Hypertension, Kawasaki Medical School, Kurashiki, Japan; 11 Saint Luke's Mid America Heart Institute, University of Missouri–Kansas City, Kansas City, MO, USA; 12 Division of Cardiology, General Hospital Murska Sobota, Murska Sobota, Slovenia; 13 Faculty of Medicine, University of Ljubljana, Ljubljana, Slovenia; 14 National Heart Centre, Singapore; 15 Duke-NUS Medical School, Singapore; 16 Kolling Institute, Royal North Shore Hospital, University of Sydney, Sydney, NSW, Australia; 17 Division of Renal Medicine, Karolinska University Hospital, Karolinska Institutet, Stockholm, Sweden; 18 Department of Renal Medicine, University College London, London, UK

**Keywords:** chronic kidney disease, methods and rationale, patient-reported outcomes, quality of life, real-world evidence

## Abstract

**Background:**

Real-world data for patients with chronic kidney disease (CKD), specifically pertaining to clinical management, metabolic control, treatment patterns, quality of life (QoL) and dietary patterns, are limited. Understanding these gaps using real-world, routine care data will improve our understanding of the challenges and consequences faced by patients with CKD, and will facilitate the long-term goal of improving their management and prognosis.

**Methods:**

DISCOVER CKD follows an enriched hybrid study design, with both retrospective and prospective patient cohorts, integrating primary and secondary data from patients with CKD from China, Italy, Japan, Sweden, the UK and the USA. Data will be prospectively captured over a 3-year period from >1000 patients with CKD who will be followed up for at least 1 year via electronic case report form entry during routine clinical visits and also via a mobile/tablet-based application, enabling the capture of patient-reported outcomes (PROs). In-depth interviews will be conducted in a subset of ∼100 patients. Separately, secondary data will be retrospectively captured from >2 000 000 patients with CKD, extracted from existing datasets and registries.

**Results:**

The DISCOVER CKD program captures and will report on patient demographics, biomarker and laboratory measurements, medical histories, clinical outcomes, healthcare resource utilization, medications, dietary patterns, physical activity and PROs (including QoL and qualitative interviews).

**Conclusions:**

The DISCOVER CKD program will provide contemporary real-world insight to inform clinical practice and improve our understanding of the epidemiology and clinical and economic burden of CKD, as well as determinants of clinical outcomes and PROs from a range of geographical regions in a real-world CKD setting.

## INTRODUCTION

Chronic kidney disease (CKD) is a major global public health problem resulting in a substantial financial and resource burden on healthcare systems worldwide according to the Global Burden of Disease (GBD) CKD Collaboration [1]. Based on a serum creatinine–based estimated glomerular filtration rate (eGFR) and urinary albumin:creatinine ratio, the Kidney Disease: Improving Global Outcomes group developed a classification system that is now widely used in clinical practice to diagnose and stratify risk in patients with CKD [2]. Using this classification, the global prevalence of identified CKD from all causes and all stages is ∼9%, equivalent to ∼700 million individuals. Since 1990, the prevalence of CKD has increased by 29% worldwide [3]. According to the most recent GBD Study, CKD is the 12th leading cause of death worldwide; in addition, CKD resulted in 36 million disability-adjusted life years, with diabetic kidney disease accounting for almost one-third of this burden [1].

Patients with CKD commonly experience an accelerated and progressive loss of kidney function and are at risk of progression to kidney failure [4]; kidney replacement therapies are lifesaving but costly. CKD is also associated with comorbidities including diabetes, hypertension, hypercholesterolemia and heart failure, as well as an increased risk of myocardial infarction and death [2].

Progressive CKD also negatively affects quality of life (QoL) and multiplies the risk of morbidity and mortality, particularly from cardiovascular causes [5]. In general, the management of patients with CKD involves preventing or slowing the progression of kidney disease, managing the complications of progressive kidney failure and preventing and managing the disease and complications in other organ systems, such as the cardiovascular and central nervous system [6–9]. In many cases, specific treatment of the underlying disease (i.e. glomerular and tubulointerstitial diseases as well as systemic diseases that affect the kidney) offers an opportunity to halt or mitigate CKD progression. For many others, measures to slow the rate of progression in patients with CKD are centered on blood pressure goals and, in patients with proteinuric disease, on reducing proteinuria with the use of angiotensin-converting enzyme inhibitors or angiotensin receptor blockers [8]. Dietary (i.e. protein restriction and a reduction in sodium intake) and lifestyle (physical activity and smoking cessation) interventions/changes may also reduce the risk of CKD progression [10]. Unfortunately, recent reports have identified that general recommendations to reduce the progression of CKD are suboptimally implemented in real-world clinical practice [11]. Revised recommended targets for blood pressure and diabetes management are now available across CKD stages [8, 9], but to what extent these recommendations will be implemented remains to be determined.

Management of CKD should also encompass the wide range of disorders and complications that develop because of the loss of kidney function. These disorders include, but are not limited to, volume overload; hyperkalemia; metabolic acidosis; difficulties in glycemic control and dyslipidemia; CKD mineral and bone disorders; cardiovascular and coronary heart diseases; signs and symptoms related to hormonal or systemic dysfunction such as anorexia, nausea, vomiting, fatigue, anemia and protein-energy wasting; as well as neuropsychiatric conditions such as depression, anxiety disorders and cognitive impairment [12–22]. The management of these complications is important in patients with CKD to reduce the risk of morbidity and mortality, reduce symptomatic burden and improve QoL [23]. Similar to what has been observed in the treatment of CKD progression, best practices recommended by guidelines and new therapies to manage these CKD complications are vastly underutilized in real-world clinical scenarios [19, 24].

Finally, there is growing interest in patient-centered care, defined as ‘care that is respectful of and responsive to individual patient preferences, needs and values’ [25]. In this horizon of important shifts in the CKD management paradigm, the inclusion of digital health solutions to capture patient-reported outcomes (PROs), including innovative ways of capturing the patients’ voice and disease awareness, is essential [26, 27].

We hypothesize that there is significant regional variation in the epidemiology and management of CKD and that there are challenges in implementing the recommended clinical practice guidelines for patient monitoring, engagement and early delivery, as well as maintenance of effective therapies. To address these issues, this study will create a global cohort that will serve as a platform to improve our understanding of the epidemiology of CKD and its associated comorbidities and complications, as well as the determinants of clinical outcomes and PROs, and will facilitate the long-term goal of improving the management and prognosis of patients with CKD from a range of geographical regions in a real-world clinical setting.

## MATERIALS AND METHODS

DISCOVER CKD is a hybrid, multinational, observational cohort study in patients with CKD comprising a retrospective patient cohort capturing secondary data from established anonymized datasets and a prospective cohort collecting primary and secondary data in patients individually recruited from participating centers in China, Italy, Japan, Sweden, the UK and the USA (ClinicalTrials.gov identifier: NCT04034992). The study aims to be descriptive by collecting data from routine clinical care.

### Study objectives

The study objectives are described in Table 1.

**Table 1.** Study objectives

Objective	Description
Primary	To construct a multinational longitudinal cohort of patients with CKD that can be used for the generation of primary and secondary real-world data in order to provide insights into the epidemiology of CKD by describing patient characteristics, disease progression, clinical outcomes, patient journey aspects, practice patterns and clinical management of CKD
Secondary	To develop country-specific descriptions of the primary objectives by different stages of CKD with a focus on the comorbidities and complications of CKD, including hyperkalemia, anemia and diabetes
Exploratory	To evaluate the monitoring and trajectories of laboratory values across different patient subgroups, as well as risk factors (covariates captured at baseline and time varying) associated with CKD progression, e.g. diabetes, and complications of CKD, e.g. hyperkalemia, anemia, diabetes and clinical outcomes

### Patient population and sample size

Adult patients from China, Italy, Japan, Sweden, the UK and the USA with a confirmed diagnosis of CKD from any cause are eligible to participate in the study. CKD will be identified by (i) documented diagnostic code (e.g. International Classification of Diseases 10) for CKD Stages 3A through to kidney failure; (ii) two consecutive eGFR measures of <75 mL/min/1.73 m^2^ [28] recorded >90 days apart (maximum 730 days) from 1 January 2008 (in Japan, eGFR will be calculated using the revised equations for eGFR from serum creatinine in Japan [29]); or (iii) a code for chronic (duration >30 days) renal replacement therapy (hemodialysis and peritoneal dialysis). Patients with a history of kidney transplant are eligible for inclusion. Exclusion criteria include current participation in any interventional trial at baseline (index; prospective cohort only), undergoing treatment for active cancer except for nonmelanoma skin cancer (prospective cohort only), life expectancy of <12 months (prospective cohort only), diagnosis of cancer on or within the 1 year prior to index (retrospective cohort only) and <1 year of medical history available prior to the index date (retrospective cohort only). The study may be supplemented with extended patient follow-up or additional countries and databases/registries.

### Prospective cohort

The initial aim is to identify and capture primary and secondary data from a minimum of 1000 (no set maximum) patients with CKD enrolled over a period of 2 years, with prospective follow-up for a minimum of 1 year. Additionally, for patients who agree to participate, patient-specific data will be captured through semistructured qualitative interviews in a randomly selected subset of ∼100 patients (~16–18 patients per country).

Sites have been selected based on an extensive list of physicians, collated with input from the managing contract research organization (PAREXEL), the study sponsor (AstraZeneca) and the DISCOVER CKD scientific committee whose members are listed in the Supplementary data, Table S1.

Eligible patients will be enrolled into the study at the time they routinely visit their physician and consent to participate. The index date will be the baseline visit; the preindex period for each patient is defined as 1 year prior to the index date, where secondary information/data on demographics, clinical assessments, laboratory values, family history, medical history, healthcare resource utilization, procedures and prescription history, as listed in Supplementary data, Table S2, will be extracted from their existing medical records and entered into the electronic case report form (eCRF) after the patient has completed the clinical visit. The number of clinical visits per year based on the current clinical practice recommendations [30] is anticipated to be as follows: CKD Stage 3A, ∼2 times per year; CKD Stage 3B, ∼2 or more times per year; CKD Stage 4, ∼3 or more times per year; and CKD Stage 5, ∼4 or more times per year (guidelines are subject to variation in different countries). This provides opportunities to follow-up with patients and capture data collected during these routine visits [30]. For a patient enrolled prospectively, the potential maximum duration in the study will be ∼3 years, which may be extended. Patients will remain enrolled until study end, study discontinuation/loss to follow-up, withdrawal from the study or death, whichever occurs first.

PROs to be collected as primary data include physical activity measured via the Rapid Assessment of Physical Activity questionnaire [31], health-related QoL via the 36-item Short Form questionnaire [32], work productivity via the Work Productivity and Activity Impairment CKD questionnaire [33] and diet via a simple 7-day food diary; other PROs, including a set of questions to collect patient symptoms, will be available on a bespoke mobile/tablet-based application. The application is available, validated and user-tested in UK English, US English, Spanish, Swedish, Italian, Japanese and Mandarin Chinese. A training diary will also be available for patients to complete. It is expected that patient questionnaires will be completed at baseline and every 6 months for as long as the patient is in the study (weekly for patient symptoms) (Supplementary data, Table S3). The full rationale for questionnaires used in DISCOVER CKD can be found in the Supplementary data.

Qualitative data specific to patients with CKD will be collected by qualitative semistructured interviews in a subset of ∼100 patients. Based on pilot work, it is anticipated that information saturation (where no new information is identified) will be achieved with this number of patients and this will also provide representation across countries [34]. These interviews will be performed by trained interviewers from a third-party vendor (Health Advances, Newton, MA, USA) with experience in CKD; the format will be either face to face or over the telephone at a time/place convenient to the patient. It is anticipated that the duration of the interviews will be 1–2 h. A recent study by James *et al.* [34] utilizing the PatientsLikeMe online community database was the pilot study for developing the interview transcript used in this study and guided decisions about which symptoms this study should capture.

### Data collection during COVID-19

As the study is collecting secondary data retrospectively, it is not designed to capture ongoing adverse events. However, per US Food and Drug Administration guidance, the study protocol has been updated to capture any delay in routine visits, the standard of care for patients with CKD and any medical event occurring between visits and/or any healthcare resource utilization between visits, specifically due to a patient contracting COVID-19 [35]. If the sites perform patient visits via telemedicine during the COVID-19 pandemic, information collected during those visits will be extracted from the medical record and entered into the eCRF as normal routine visits by the sites.

### Retrospective cohort

**FIGURE 1:**
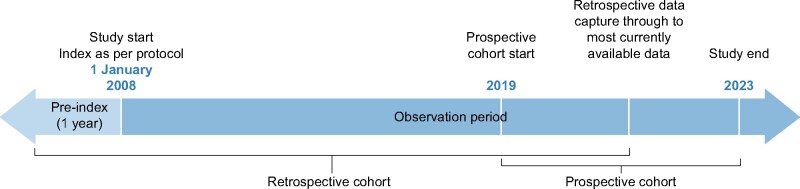
Study period.

The retrospective patient cohort includes secondary data extracted from established anonymized datasets [e.g. electronic health record (EHR) or claims databases that AstraZeneca has licensed for internal analysis, or through external collaborations] and harmonized into a common data model. These may include, but are not limited to, those listed in Table 2 [36–42]. The retrospective cohort will capture incident patients with CKD beginning 1 January 2008, through the most currently available data (Figure 1). Variables to be collected are outlined in Supplementary data, Table S2.

**Table 2.** Established databases currently included in DISCOVER CKD

Database name	Country	Database type	Coverage	References
TriNetX	USA	EHR	Inpatient and outpatient	Topaloglu U *et al. JCO Clin Cancer Informatics.* 2018; 2: 1–10 [36]
Explorys (LCED)	USA	EHR and claims	Inpatient and outpatient	https://www.ibm.com/watson-health/about/explorys [37]
J-MDV	Japan	EHR and claims	Inpatient and outpatient	Tanaka S *et al. J Pharm Heal Care Sci.* 2015; 1: 16 [38]
CPRD GOLD	UK	EHR	Primary care, inpatient and outpatient, emergency room	Herrett E *et al. Int J Epidemiol*. 2015; 44: 827–836 [39]
J-CKD (Kawasaki Medical School)	Japan	EHR	Inpatient and outpatient	Nakagawa N *et al*. *Sci Rep.* 2020; 10: 7351 [40]
SCREAM	Sweden	EHR	Inpatient and outpatient	Runesson B *et al*. *Clin Kidney J*. 2016; 119–127 [41]
DOPPS	USA	EHR	Hemodialysis	Pisoni RL *et al*. *Am J Kidney Dis*. 2004; 45: 8 [42]

LCED, Limited Claims and Electronic Health Records Database.

### Statistical analysis

**FIGURE 2:**
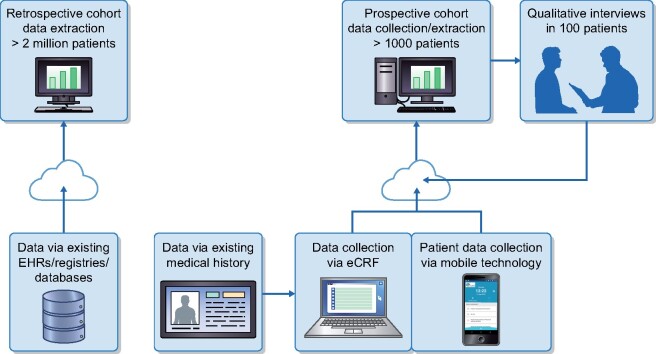
Retrospective cohort and prospective cohort data collection.

Analyses will be conducted separately for prospective and retrospective CKD cohorts and then in the aggregate by combining prospective and retrospective data, to the extent possible (e.g. comparing index characteristics), at the end of the study. A master statistical analysis plan has been created that details the methods that can be utilized to answer the study objectives; prior to each specific analysis, sample size will be assessed to ensure that there is sufficient precision for key estimates. Site characteristics will be ascertained (e.g. primary versus tertiary care, urban versus rural, academic versus nonteaching, etc.) and used in the analyses for adjustment, stratification or sensitivity analysis accordingly. Extraction of clinical data will be conducted in accordance with country-specific data privacy laws, governance and patient consent (where applicable). Ultimately data collected in DISCOVER CKD will be harmonized and integrated to allow pooled patient-level analysis or aggregated/meta analysis (Figure 2).

## DISCUSSION

Currently there is a need to understand current practice patterns and patient perspectives related to the management of CKD progression and its complications. Collecting patient data from diverse regions will provide regional insights into real-world practice patterns and clinical management of CKD in participating countries. DISCOVER CKD aims to address epidemiological gaps and to understand the disease trajectory of patients with CKD from different geographies, complementing other multinational [43, 44], national and regional CKD/kidney failure cohort studies [41, 45–49] and registries, including initiatives to collect data for patients receiving dialysis [42, 50–53].

Study design, sample size, profile of patients and type of data available vary widely, and comparisons across studies are challenging. The major strengths of this study comprise geographic representation by the inclusion of patients from different countries and healthcare settings, the large sample size, the collection of real-world, routine care data on the clinical management of patients with CKD, as well as capturing information about CKD effects beyond the kidney itself. We will include complications and diseases of other organ systems, with specific focus on the cardiovascular system and diabetes. Also unique to this study is the linkage of clinical data with patient-reported data, which we will amalgamate to create a richer database to further aid our understanding of disease progression and the long-term factors associated with CKD development and PROs. By examining the clinical management of patients across and within multiple countries, we will be able to provide important insights to support the development of public health awareness and disease management, especially in countries where these data do not yet exist or have not been examined due to limitations in data capture. Additionally, by having broad inclusion criteria for patients with CKD, i.e. inclusion of multiple stages of CKD as opposed to only advanced CKD, we will be able to generalize our findings to the wider population of patients with CKD. Also, by using a longitudinal cohort study design, we will be able to examine disease trajectories and the factors associated with disease stability and disease progression, including the speed of progression.

DISCOVER CKD is characterized by an innovative approach to data collection across different regions and countries using a cross-link between clinical, laboratory and diagnostic methods (e.g. imaging) and a collection of a broad array of clinical outcomes and PROs [54, 55]. The development and implementation of user-friendly and electronic collection of data should improve compliance with data capture and allow for the generation of a database with unique information, particularly when aligned with the combination of retrospective EHR collection and data linkage. With the prospective collection of a broad list of outcomes, together with the increasing performance of big data storage, organization and analysis, DISCOVER CKD provides a unique opportunity to fill important gaps in understanding the journey of patients with CKD. Collection of insights via patient interviews will complement medical and PRO data collection, thereby enabling a more in-depth understanding of the patients’ perspectives and experiences of the disease. In fact, the DISCOVER CKD research strategy is aligned with a growing focus on the importance of a patient’s engagement and self-management with the support of various mobile phone apps [27, 49]. The project aims to address the limited availability of validated technology, as well as the missing robust evidence on the role of these new digital health technologies in CKD. The results of this project will address the scientific community’s knowledge gaps about the real impact of how effectively digital health solutions potentially improve patient engagement and disease awareness, as well as how patient-generated data, including PROs, can contribute to individualized care and therefore improve outcomes and patient QoL. There are important gaps in knowledge and current needs related to patient-specific data such as PROs; although a patient-centered approach is increasingly recognized, it is still underutilized. DISCOVER CKD intends to give the patient a voice to provide their experiences with CKD and, through qualitative interviews and PROs collection, to identify gaps in treatment, care pathways and novel endpoints to enhance patient dialog.

In view of the current pandemic, DISCOVER CKD has amended the study protocol to detect and verify the potential impact of COVID-19 on CKD management, as has been reported [35]. Witnessing the major changes that have affected daily life globally during the pandemic, several effects can be expected for patients with CKD [35]. In this respect, DISCOVER CKD has the potential to identify gaps in disease management, and to suggest strategies to maintain current standards of good clinical practice for patients with CKD.

There are potential limitations that need to be considered. First, in some instances, patient data included in the retrospective cohort may theoretically be duplicated across databases from the same country. However, that likelihood is small, as a CKD patient at Stage 3A or higher is likely to have a clinical visit at least 2–3 times per year. Therefore the required period of 1-year registration/medical history prior to the index date reduces the likelihood of duplicating patient data. Second, although we aim to collect data systematically and via different methods (as described above), heterogeneity in the coding systems used and the clinical management of patients (e.g. nephrology, general practice, cardiology and endocrinology) might result in differences in the quality of collected data. Nevertheless, because this study will reflect how data are collected in routine practice in the real world, where there are many differences in healthcare settings and treatment choices, these data will provide real-world insights on actual within- and between-country differences, as well as differences between primary and secondary care settings, thereby providing data that are complementary to data collected in randomized clinical trials and can be used to assess the impact these settings have on clinical outcomes. Third, EHR and claims data are not collected for research purposes and may therefore lack information on specific covariates that may be associated with CKD. However, these data will offer important variables, including lab values, as well as provide crucial disease insights and help to contextualize study findings. Missing variables and missing information within variables will be assessed. Fourth, patients may incorrectly recall self-reported information, leading to differential recall bias; for example, incorrect recall of diet or physical activity leading to over-/underestimation of inferences. Finally, the indication for treatment and side effects of therapies may not be recorded. Despite this, treatment indication can be inferred when a product has a single indication and little to no off-label use.

The DISCOVER CKD program will establish multiregional retrospective and prospective cohorts of patients with CKD, integrating comprehensive, high-quality primary and secondary longitudinal data, including qualitative assessment of patients’ perspectives through interviews. This study will provide contemporary real-world insights during the pre-, current and post-COVID-19 pandemic landscape to not only inform clinical practice, but also to improve our understanding of the epidemiology, clinical burden and economic burden of CKD and its associated comorbidities and complications, as well as the determinants of clinical outcomes and PROs from a range of geographical regions in a real-world CKD setting.

## Supplementary Material

sfab046_supplementary_data
