# Supplementary material for: Methods and rationale of the DISCOVER CKD global observational study
Source: Clin Kidney J. 2021 Apr 11;14(6):1570–8. doi: 10.1093/ckj/sfab046 (PMC8264307; doi:10.1093/ckj/sfab046)
Supplement: sfab046_supplementary_data [file sfab046_supplementary_data.docx]

Table of Contents

[Description of Questionnaires 2](#_Toc57210370)

[References 4](#_Toc57210371)

[Supplementary Table S1. Discover CKD scientific committee members 5](#_Toc57210372)

[Supplementary Table S2. Study variables (primary and secondary) planned to be collected/extracted as part of routine clinical care 6](#_Toc57210373)

[Supplementary Table S3. Prospective patient-specific data collection 20](#_Toc57210374)

# Description of Questionnaires

Many of the questionnaires used to assess health-related quality of life in patients with chronic kidney disease (CKD)/kidney failure have the disadvantages of being too long for routine use, not having been well-validated, or only being relevant for a specific group of patients with CKD, such as patients on dialysis. The reliability and validity of the Short Form (SF)-36 questionnaire have been well assessed, and normative data exist for English-speaking populations. It has also been shown to be valid and acceptable to patients with kidney failure [1]. The SF-36 form has also been translated and validated in several other languages. Similarly, physical inactivity is a long‑standing clinical problem among pre-dialysis patients with CKD, as well as in patients with kidney failure on dialysis treatment. It contributes to disability and poor nutritional status, which are associated with increased morbidity and mortality risk. The Rapid Assessment of Physical Activity questionnaire is a highly accessible, simple, self-administered and not time‑consuming questionnaire used to measure the physical activity of an individual. It has all the limits of a self‑reporting questionnaire but may be very useful for extensive routine implementation in daily clinical practice. It has also been favourably tested as a telephone‑based questionnaire [2]. The Work Productivity and Activity Impairment CKD questionnaire was created as a patient-reported quantitative assessment of the amount of absenteeism, presenteeism and daily activity impairment attributable to general health or a specific health problem. Therefore, use of this questionnaire during the study will help identify work productivity losses among CKD sufferers due to loss of kidney function and underlying comorbidities including anaemia. It can also provide a basis for further research to explore whether work productivity loss resulting from CKD can be better managed by implementing early detection and chronic care management strategies. The 7-day food diary will help describe any changes made to a patient’s diet as a result of CKD, which may include limiting fluids, salt, potassium, phosphorous and other electrolytes, or eating a low-protein diet, while ensuring that patients are getting enough calories [3]. Lastly, collecting weekly symptom information will aid understanding of the changes and severity of symptoms commonly reported by patients with CKD [4].

# References

1. Wight JP, Edwards L, Brazier J *et al.* The SF36 as an outcome measure of services for end stage renal failure. *Qual Health Care* 1998; 7: 209-221

2. Cupisti A, D'Alessandro C, Finato V *et al.* Assessment of physical activity, capacity and nutritional status in elderly peritoneal dialysis patients. *BMC Nephrol* 2017; 18: 180

3. Ikizler TA, Burrowes JD, Byham-Gray LD *et al.* KDOQI Clinical Practice Guideline for Nutrition in CKD: 2020 Update. *Am J Kidney Dis* 2020; 76: S1-S107

4. James G, Nyman E, Fitz-Randolph M *et al.* Characteristics, Symptom Severity, and Experiences of Patients Reporting Chronic Kidney Disease in the PatientsLikeMe Online Health Community: Retrospective and Qualitative Study. *J Med Internet Res* 2020; 22: e18548

Supplementary Table S1. DISCOVER CKD scientific committee members

| **Name** | **Affiliation** |
| --- | --- |
| Hiddo Heerspink | Department of Clinical Pharmacy and Pharmacology, University of Groningen, Groningen, the Netherlands |
| Peter Stenvinkel | Division of Renal Medicine, Karolinska University Hospital, Karolinska Institutet, Stockholm, Sweden |
| Mikhail Kosiborod | Saint Luke's Mid America Heart Institute, University of Missouri-Kansas City, Kansas City, USA |
| Carolyn S.P. Lam | National Heart Centre, Singapore & Duke-National University of Singapore, Singapore |
| Naoki Kashihara | Kawasaki Medical School, Kurashiki, Japan |
| Eiichiro Kanda | Kawasaki Medical School, Kurashiki, Japan |
| David Wheeler | Department of Renal Medicine, University College London, London, UK. |
| Carol Pollock | Kolling Institute, Royal North Shore Hospital, University of Sydney, Sydney, NSW, Australia |
| Mitja Lainscak | Division of Cardiology, General Hospital Murska Sobota, Murksa Sobota, Slovenia |
| Roberto Pecoits-Filho | School of Medicine, Pontifícia Universidade Católica do Paraná, Curitiba, Paraná, Brazil, Arbor Research Collaborative for Health, Ann Arbor, MI, USA |
| Steven Fishbane | Division of Nephrology, Zucker School of Medicine at Hofstra/Northwell, Hempstead, NY, USA |
| Juan Jesus Carrero | Department of Medical Epidemiology and Biostatistics, Karolinska Institutet, Stockholm, Sweden |

Supplementary Table S2. Study variables (primary and secondary) planned to be collected/extracted as part of routine clinical care

| **Variable** | **Collection/ extraction point** | **Prospective cohort^a^** | **Retrospective cohort^b^** | **Prospective data extracted from^c^** | **Retrospective data extracted from** |
| --- | --- | --- | --- | --- | --- |
| Demographics | | | | | |
| Specialty of enrolling physician (cardiology, nephrology, general practitioner, other) | Baseline | Yes | No | HCP | NA |
| Sex | Baseline | Yes | Yes | HR | EHR |
| Age at enrolment | Baseline | Yes | Yes | HR | EHR |
| Date of visit | Baseline/routine visit | Yes | No | HR | EHR |
| Race | Baseline | Yes | Yes | HR | EHR |
| Employment status | Baseline/routine visit | Yes | Yes | HR | EHR |
| Marital status | Baseline/routine visit | Yes | Yes | HR | EHR |
| Geographic location (site country) | Baseline | Yes | Yes | HCP | EHR |
| Health insurance coverage | Baseline/routine visit | Yes | Yes | HR | EHR |
| Smoking status | Baseline/routine visit | Yes | Yes | HR | EHR |
| Alcohol consumption (units per week) | Baseline/routine visit | Yes | Yes | HR | EHR |
| Clinical assessment | | | | | |
| Height | Baseline | Yes | Yes | HR | EHR |
| Weight | Baseline/routine visit | Yes | Yes | HR | EHR |
| Body mass index (calculated) | Baseline/routine visit | Yes | Yes | HR | EHR |
| Systolic blood pressure | Baseline/routine visit | Yes | Yes | HR | EHR |
| Diastolic blood pressure | Baseline/routine visit | Yes | Yes | HR | EHR |
| Heart rate | Baseline/routine visit | Yes | Yes | HR | EHR |
| Laboratory values (includes dates obtained) | | | | | |
| Estimated glomerular filtration rate | Baseline/routine visit | Yes | Yes | HR | EHR |
| Serum bicarbonate | Baseline/routine visit | Yes | Yes | HR | EHR |
| Cystatin C | Baseline/routine visit | Yes | Yes | HR | EHR |
| Vitamin D (defined as 1,25 or 25 hydroxy vitamin D) | Baseline/routine visit | Yes | Yes | HR | EHR |
| Creatinine | Baseline/routine visit | Yes | Yes | HR | EHR |
| Albumin | Baseline/routine visit | Yes | Yes | HR | EHR |
| Haemoglobin A1c or fasting plasma glucose | Baseline/routine visit | Yes | Yes | HR | EHR |
| Urine glucose | Baseline/routine visit | Yes | Yes | HR | EHR |
| Urine albumin/creatinine ratio (also known as ACR or UPCR or reagent strip qualitative recording) | Baseline/routine visit | Yes | Yes | HR | EHR |
| Uric acid | Baseline/routine visit | Yes | Yes | HR | EHR |
| Pro-N-terminal-brain natriuretic peptide | Baseline/routine visit | Yes | Yes | HR | EHR |
| Brain natriuretic peptide | Baseline/routine visit | Yes | Yes | HR | EHR |
| CRP (also high sensitivity CRP) | Baseline/routine visit | Yes | Yes | HR | EHR |
| Total cholesterol | Baseline/routine visit | Yes | Yes | HR | EHR |
| LDL-C | Baseline/routine visit | Yes | Yes | HR | EHR |
| HDL-C | Baseline/routine visit | Yes | Yes | HR | EHR |
| LDL-C/HDL-C ratio (calculated) | Baseline/routine visit | Yes | Yes | HR | EHR |
| Triglycerides | Baseline/routine visit | Yes | Yes | HR | EHR |
| Calcium | Baseline/routine visit | Yes | Yes | HR | EHR |
| Potassium | Baseline/routine visit | Yes | Yes | HR | EHR |
| Sodium | Baseline/routine visit | Yes | Yes | HR | EHR |
| Phosphate/phosphorous | Baseline/routine visit | Yes | Yes | HR | EHR |
| Ferritin | Baseline/routine visit | Yes | Yes | HR | EHR |
| Haemoglobin | Baseline/routine visit | Yes | Yes | HR | EHR |
| Haematocrit | Baseline/routine visit | Yes | Yes | HR | EHR |
| Platelets | Baseline/routine visit | Yes | Yes | HR | EHR |
| Reticulocyte count | Baseline/routine visit | Yes | Yes | HR | EHR |
| Eosinophil count | Baseline/routine visit | No | Yes | NA | EHR |
| Mean corpuscular volume | Baseline/routine visit | Yes | Yes | HR | EHR |
| White blood cell count | Baseline/routine visit | Yes | Yes | HR | EHR |
| Transferrin saturation | Baseline/routine visit | Yes | Yes | HR | EHR |
| Total iron binding capacity | Baseline/routine visit | Yes | Yes | HR | EHR |
| Iron | Baseline/routine visit | Yes | Yes | HR | EHR |
| Red blood cell count | Baseline/routine visit | Yes | Yes | HR | EHR |
| Parathyroid hormone | Baseline/routine visit | Yes | Yes | HR | EHR |
| Medical/procedural history/outcomes (includes dates diagnosed/obtained, diagnostic code, and classification system used) | | | | | |
| Known/suspected cause of CKD | Baseline | Yes | Yes | HR | EHR |
| Hyperkalaemia, defined as mild: K+ >5.0–5.4 mmol/L, moderate; K+ 5.5–5.9 mmol/L and severe K+ >6.0 mmol/L | Baseline/routine visit | Yes | Yes | HR | EHR |
| Anaemia, defined by the World Health Organization as haemoglobin levels <12.0 g/dL in women and <13.0 g/dL in men (type) | Baseline/routine visit | Yes | Yes | HR | EHR |
| Blood transfusion | Baseline/routine visit | Yes | Yes | HR | EHR |
| Diabetes (type to be specified) | Baseline/routine visit | Yes | Yes | HR | EHR |
| Oedema | Baseline/routine visit | Yes | Yes | HR | EHR |
| Heart failure (to include New York Heart Association class and ejection fraction [heart failure with preserved ejection fraction/heart failure with reduced ejection fraction]) | Baseline/routine visit | Yes | Yes | HR | EHR |
| Coronary heart disease | Baseline/routine visit | Yes | Yes | HR | EHR |
| Gout | Baseline/routine visit | Yes | Yes | HR | EHR |
| Coronary revascularization (including percutaneous coronary intervention, thrombolytic therapy and coronary artery bypass graft) | Baseline/routine visit | Yes | Yes | HR | EHR |
| Angina pectoris | Baseline/routine visit | Yes | Yes | HR | EHR |
| Myocardial infarction | Baseline/routine visit | Yes | Yes | HR | EHR |
| Stroke | Baseline/routine visit | Yes | Yes | HR | EHR |
| Bleeding | Baseline/routine visit | Yes | Yes | HR | EHR |
| Transient ischemic attack | Baseline/routine visit | Yes | Yes | HR | EHR |
| Peripheral arterial diseases | Baseline/routine visit | Yes | Yes | HR | EHR |
| Venous thromboembolism (deep vein thrombosis/pulmonary embolism) | Baseline/routine visit | Yes | Yes | HR | EHR |
| Arteriovenous fistula embolization (haemodialysis) | Baseline/routine visit | Yes | Yes | HR | EHR |
| Atrial fibrillation/flutter/ventricular tachycardia | Baseline/routine visit | Yes | Yes | HR | EHR |
| Bradycardia | Baseline/routine visit | Yes | Yes | HR | EHR |
| Atrioventricular block | Baseline/routine visit | Yes | Yes | HR | EHR |
| Albuminuria | Baseline/routine visit | Yes | Yes | HR | EHR |
| Acute kidney injury | Baseline/routine visit | Yes | Yes | HR | EHR |
| Polycystic kidney disease | Baseline/routine visit | Yes | Yes | HR | EHR |
| Retinopathy | Baseline/routine visit | Yes | Yes | HR | EHR |
| Diabetic neuropathy | Baseline/routine visit | Yes | Yes | HR | EHR |
| Hypertension | Baseline/routine visit | Yes | Yes | HR | EHR |
| Hyperlipidaemia | Baseline/routine visit | Yes | Yes | HR | EHR |
| Infection in previous 12 months | Baseline/routine visit | Yes | Yes | HR | EHR |
| Pancreatitis | Baseline/routine visit | Yes | Yes | HR | EHR |
| Respiratory disease (specify) | Baseline/routine visit | Yes | Yes | HR | EHR |
| Chronic obstructive pulmonary disease | Baseline/routine visit | Yes | Yes | HR | EHR |
| Systemic lupus erythematosus/lupus | Baseline/routine visit | Yes | Yes | HR | EHR |
| Human immunodeficiency virus | Baseline/routine visit | Yes | Yes | HR | EHR |
| Dialysis (specify type, vintage, modality, access type, prescription, frequency, duration and adequacy) | Baseline/routine visit | Yes | Yes | HR | EHR |
| Percutaneous transluminal angioplasty/ablation | Baseline/routine visit | Yes | Yes | HR | EHR |
| Cystic fibrosis | Baseline/routine visit | No | Yes | NA | EHR |
| Haemochromatosis | Baseline/routine visit | No | Yes | NA | EHR |
| Polycystic ovary syndrome | Baseline/routine visit | No | Yes | NA | EHR |
| Cushing’s syndrome | Baseline/routine visit | No | Yes | NA | EHR |
| Glucagonoma | Baseline/routine visit | No | Yes | NA | EHR |
| Lupus nephritis | Baseline/routine visit | No | Yes | NA | EHR |
| Anti-neutrophil cytoplasmic antibody nephritis | Baseline/routine visit | No | Yes | NA | EHR |
| Hypoglycaemia | Baseline/routine visit | No | Yes | NA | EHR |
| Hyperglycaemia | Baseline/routine visit | No | Yes | NA | EHR |
| Immunoglobulin A nephropathy | Baseline/routine visit | No | Yes | NA | EHR |
| Cancer | Baseline/routine visit | Yes | Yes | HR | EHR |
| Death (cause of) | Baseline/routine visit | Yes | Yes | HR | EHR |
| Family history | | | | | |
| Do you have a family history of CKD? | Baseline | Yes | Yes | HR | EHR |
| Do you have a family history of dialysis? | Baseline | Yes | Yes | HR | EHR |
| Do you have a family history of diabetes | Baseline | Yes | Yes | HR | EHR |
| Do you have a family history of anaemia? | Baseline | Yes | Yes | HR | EHR |
| Do you have a family history of hyperkalaemia? | Baseline | Yes | Yes | HR | EHR |
| Healthcare resource utilization (includes dates, codes and reason) | | | | | |
| Hospitalization (including type of care unit, number of days, reason/diagnosis code, medical code and readmissions) | Baseline/routine visit | Yes | Yes | HR | EHR |
| Outpatient visit (including type of care unit, reason/diagnosis code, medical code and readmissions) | Baseline/routine visit | Yes | Yes | HR | EHR |
| Intensive care unit stay (including type of care unit, number of days, reason/diagnosis code, medical code and readmissions) | Baseline/routine visit | Yes | Yes | HR | EHR |
| Emergency room visit (including type of care unit, number of days, reason/diagnosis code, medical code and readmissions) | Baseline/routine visit | Yes | Yes | HR | EHR |
| Required use of an ambulance (including reason and medical code) | Baseline/routine visit | Yes | Yes | HR | EHR |
| Discharge status (where the patient was sent [e.g home], reason and medical code) | Baseline/routine visit | Yes | Yes | HR | EHR |
| Referral to specialist(s) (including reason and medical code) | Baseline/routine visit | Yes | Yes | HR | EHR |
| Referral/use of other supportive care (including reason) | Baseline/routine visit | Yes | Yes | HR | EHR |
| Procedures | | | | | |
| Dialysis (specify type, vintage, modality, access type, prescription, frequency, duration and adequacy) | Baseline/routine visit | Yes | Yes | HR | EHR |
| Transplant (type) | Baseline/routine visit | Yes | Yes | HR | EHR |
| Imaging/magnetic resonance imaging/computed tomography/ultrasound | Baseline/routine visit | Yes | Yes | HR | EHR |
| Home oxygen therapy | Baseline/routine visit | Yes | Yes | HR | EHR |
| IV iron transfusion | Baseline/routine visit | Yes | Yes | HR | EHR |
| Dialysis catheter insertion | Baseline/routine visit | Yes | Yes | HR | EHR |
| Arteriovenous fistula creation | Baseline/routine visit | Yes | Yes | HR | EHR |
| Arteriovenous graft creation | Baseline/routine visit | Yes | Yes | HR | EHR |
| Electrocardiogram (to include heart rate, PQ/QT interval, etc.) | Baseline/routine visit | Yes | Yes | HR | EHR |
| Self-blood glucose monitoring strips | Baseline/routine visit | No | Yes | NA | EHR |
| Pancreatectomy | Baseline/routine visit | No | Yes | NA | EHR |
| Prescriptions (includes drug names, reason for prescription, route of administration, date, dose and reason for discontinuation) | | | | | |
| Angiotensin-converting enzyme inhibitors | Baseline/routine visit | Yes | Yes | HR | EHR |
| Angiotensin II receptor blockers | Baseline/routine visit | Yes | Yes | HR | EHR |
| Mineralocorticoid receptor antagonists | Baseline/routine visit | Yes | Yes | HR | EHR |
| Angiotensin receptor-neprilysin inhibitors | Baseline/routine visit | Yes | Yes | HR | EHR |
| Omega-3 fatty acids | Baseline/routine visit | Yes | Yes | HR | EHR |
| Nitrates | Baseline/routine visit | Yes | Yes | HR | EHR |
| Calcium channel blockers, dihydropyridine, non- dihydropyridine | Baseline/routine visit | Yes | Yes | HR | EHR |
| Beta blockers | Baseline/routine visit | Yes | Yes | HR | EHR |
| Alpha blockers | Baseline/routine visit | Yes | Yes | HR | EHR |
| Sodium zirconium cyclosilicate | Baseline/routine visit | Yes | Yes | HR | EHR |
| Roxadustat | Baseline/routine visit | Yes | Yes | HR | EHR |
| Dapagliflozin | Baseline/routine visit | Yes | Yes | HR | EHR |
| Canagliflozin | Baseline/routine visit | Yes | Yes | HR | EHR |
| Empagliflozin | Baseline/routine visit | Yes | Yes | HR | EHR |
| Dipeptidyl peptidase-4 inhibitors | Baseline/routine visit | Yes | Yes | HR | EHR |
| Metformin | Baseline/routine visit | Yes | Yes | HR | EHR |
| Other oral anti-diabetes, e.g. sulfonylureas, thiazolidinediones, biguanides and alpha-glucosidase inhibitors | Baseline/routine visit | Yes | Yes | HR | EHR |
| Insulin | Baseline/routine visit | Yes | Yes | HR | EHR |
| Glucagon-like peptide-1 receptor agonists | Baseline/routine visit | Yes | Yes | HR | EHR |
| Other sodium-glucose cotransporter-2 inhibitors | Baseline/routine visit | Yes | Yes | HR | EHR |
| Erythropoiesis-stimulating agent | Baseline/routine visit | Yes | Yes | HR | EHR |
| Iron (oral and IV) | Baseline/routine visit | Yes | Yes | HR | EHR |
| Sodium polystyrene sulfonate | Baseline/routine visit | Yes | Yes | HR | EHR |
| Calcium polystyrene sulfonate | Baseline/routine visit | Yes | Yes | HR | EHR |
| Patiromer | Baseline/routine visit | Yes | Yes | HR | EHR |
| Statin | Baseline/routine visit | Yes | Yes | HR | EHR |
| Loop diuretic | Baseline/routine visit | Yes | Yes | HR | EHR |
| Thiazide diuretic | Baseline/routine visit | Yes | Yes | HR | EHR |
| Nonsteroidal anti-inflammatory drugs | Baseline/routine visit | Yes | Yes | HR | EHR |
| Histamine 2 blockers | Baseline/routine visit | Yes | Yes | HR | EHR |
| Proton-pump inhibitors | Baseline/routine visit | Yes | Yes | HR | EHR |
| Steroid/immunosuppressant | Baseline/routine visit | Yes | Yes | HR | EHR |
| Aspirin | Baseline/routine visit | Yes | Yes | HR | EHR |
| Antiplatelet agents (including potency) | Baseline/routine visit | Yes | Yes | HR | EHR |
| Anticoagulants | Baseline/routine visit | Yes | Yes | HR | EHR |
| Others antihyperlipidemic treatment | Baseline/routine visit | Yes | Yes | HR | EHR |
| Fibrates | Baseline/routine visit | Yes | Yes | HR | EHR |
| Niacin | Baseline/routine visit | Yes | Yes | HR | EHR |
| PCSK9 inhibitors | Baseline/routine visit | Yes | Yes | HR | EHR |
| Sodium bicarbonate | Baseline/routine visit | Yes | Yes | HR | EHR |
| Patient-specific data | | | | | |
| Health related quality of life – Short Form-36 questionnaires | Baseline and every 6 months | Yes | No | Recorded by patient | NA |
| Physical activity – Rapid Assessment of Physical Activity questionnaire | Baseline and every 6 months | Yes | No | Recorded by patient | NA |
| Other patient-reported outcomes, including a set of questions to collect patient symptoms | Baseline and weekly | Yes | No | Recorded by patient | NA |
| Diet – simple food diary | Collected for a 7-day period at baseline and then every 6 months | Yes | No | Recorded by patient | NA |
| Work productivity – Work Productivity and Activity Impairment questionnaire | Baseline and every 6 months | Yes | No | Recorded by patient | NA |

ACR, albumin to creatinine ratio; CKD, chronic kidney disease; CRP, C-reactive protein; EHR, electronic health record; HCP, healthcare professional; HDL-C, high-density lipoprotein cholesterol; HR, health record; IV, intravenous; LDL-C, low-density lipoprotein cholesterol; NA, not applicable; PCSK9, proprotein convertase subtilisin/kexin type 9; UPCR, urine protein to creatinine ratio.

^a^Prospective cohort data collection/extraction will also include pre-index medical history available to the enrolling physician.

^b^To include information available pre-index.

^c^Where data will be manually extracted from and manually entered into the eCRF after the clinical visit (except for patient-specific data recorded by patient).

Note: Variables will be manually collected/extracted as defined in the electronic case report form.

Data from different units will be harmonized into one preferred unit.

The above list may include variables not part of routine care in some countries; however, if this is missing data the study anticipates ascertaining whether the data is missing due to not being entered or simply because it is not part of routine clinical practice. This will be useful to understand clinical management.

Missingness of data will also be assessed to determine completeness of data; this is also useful to understand clinical management across counties.

Where possible and to reduce ‘noise’ in the data, we will manually collect/extract data specific to the nephrology setting to, as best as possible, portray the true clinical management and journey of patients with CKD.

Supplementary Table S3. Prospective patient-specific data collection

| **Data collection** | **Baseline (index)** | **Data collection in Years 1, 2 and 3^a^** | |
| --- | --- | --- | --- |
| Timeframe for collection | Consent day or within 14 days after | 6-monthly collection ± 30 days | 12-monthly collection ± 30 days |
| Patient symptom information (mobile phone/tablet application^b^); fatigue, pruritis (itching), problems with sleep, numbness or tingling, chest pain, shortness of breath, nausea, muscle cramping, depressed or unhappy feelings, anxious or worried feelings, feeling of overall health today | Yes | Yes | Yes |
| Physical activity questionnaires (mobile phone/tablet application) – Rapid Assessment of Physical Activity | Yes | Yes | Yes |
| Health related quality of life questionnaires (mobile phone/tablet application) – Short Form-36 health survey | Yes | Yes | Yes |
| Work productivity questionnaires (mobile phone/tablet application) – Work Productivity and Activity Impairment questionnaire | Yes | Yes | Yes |
| Diet (mobile phone/tablet application) – simple food diary^c^ | Yes | Yes | Yes |

^a^For patient-specific data, patients can choose to complete the information listed in the table during their clinical appointment or in their own time via the mobile phone/tablet application (approximately every 6 months and weekly for patient symptoms regardless of frequency of routine visit).

^b^Collected weekly via the mobile phone/tablet application.

^c^To be collected for a 7-day period approximately every 6 months.
